# The Effect of Repeated Pressing on the Flexural Strength, Color Stability, Vickers Hardness, and Surface Topography of Heat-Pressed Lithium Disilicate

**DOI:** 10.3390/ma15196787

**Published:** 2022-09-30

**Authors:** Tariq S. AbuHaimed, Saeed J. Alzahrani, Sami A. Farsi, Lulwa E. AL-Turki, Maher S. Hajjaj

**Affiliations:** 1 Department of Restorative Dentistry, Faculty of Dentistry, King Abdulaziz University, Jeddah 21589, Saudi Arabia; 2 King Fahad Hospital, Ministry of Health, Jeddah 23325, Saudi Arabia; 3 Department of Oral and Maxillofacial Prosthodontics Dentistry, Faculty of Dentistry, King Abdulaziz University, Jeddah 22254, Saudi Arabia

**Keywords:** ceramic, heat-pressing, esthetic

## Abstract

The aim of this study was to investigate the effect of repressing leftover heat-pressed lithium disilicate material on its mechanical and optical properties. A lithium disilicate ingot (IPS e.max^®^ Press, IvoclarVivadent, Schaan, Liechtenstein) shade (A1) low translucency was first heat-pressed to yield ceramic bars and disks. Then, the second and third presses were fabricated from the leftovers of the previous pressing cycles. A total of 36 bars and 15 disk specimens were fabricated and divided into three groups according to the number of pressing cycles (n = 12 bars and n = 5 disks): P1: first press (control), P2: second press, and P3: third press. The specimens were tested for flexural strength, color change, Vickers hardness, and surface topography under scanning electron microscopy. One-way ANOVA testing was used to evaluate flexural strength and hardness, while an independent t-test was performed to evaluate color change. There was no significant difference in flexural strength as the number of heat-pressed cycles increased (*p* = 0.283). Similarly, there was no significant difference in the microhardness values between all groups (*p* = 0.220). The overall color change ∆E between P1–P2 and P1–P3 were 2.01 and 2.14, respectively. The SEM images showed evenly distributed and densely packed lithium disilicate crystals in the P1 group. However, larger and less densely packed crystals were noticeable in P2 and P3. The IPS e.max Press could be repressed up to two times without an adverse effect on mechanical properties or color stability. These results may support the reuse of pressed lithium disilicate for economical purposes, but further clinical evaluation should be conducted to confirm these findings.

## 1. Introduction

Dental ceramics can be classified according to their microstructure into glass-based (silica), glass-based (silica) with fillers, crystalline-based with glass fillers (alumina, zirconia), or polycrystalline solids (zirconia and alumina) [1,2]. Additionally, they can be classified according to their fabrication methods, namely, conventional powder/liquid, slip casting, heat-pressed, or CAD/CAM systems. The mechanical and optical properties of each system are dependent on their inherent microstructure and fabrication methods [3].

Heat-pressed glass ceramics systems have been in the market for over three decades and have remained one of the most preferred dental ceramics in a wide range of clinical applications [4,5]. The first generation of this type of glass ceramic was IPS Empress (Ivovlar Vivadent), a leucite-filled heat-pressed esthetic glass ceramic [2]. The introduction of lithium disilicate pressable glass ceramics, Empress 2 (IPS Empress 2, Ivoclar Vivadent), with improved mechanical properties allowed the production of three-unit anterior fixed dental prosthesis. Lithium disilicate glass ceramics are heat-pressed glass ceramic composed of a glassy phase with lithium phosphate nanophase (Li_3_PO_4_) precipitate for the nucleation of lithium metasilicate, consequently forming lithium disilicate needle-like crystals. Further development led to the introduction of IPS e.max Press (Ivoclar Vivadent), composed of 70% lithium disilicate crystals dispersed in a glass matrix, which offered a higher degree of resistance to crack propagation and ultimately improved the flexural strength [6]. Due to the lack of metallic components in the microstructure and low refractive index, lithium disilicate offers excellent esthetics and high translucency that mimics the natural teeth [7]. These favorable properties were documented by satisfactory long-term clinical data of up to 10 years [4,8,9], The heat-pressed fabrication method utilizes the lost wax technique. Further development introduced IPS e.max CAD (Ivoclar Vivadent), a milled lithium disilicate glass ceramic, which offered the advantage of ease and speed of fabrication.

Both pressing and milling fabrication techniques leave a considerable quantity of leftover material. However, the pressing technique may offer the advantage of reusing what is usually discarded. After pressing, the leftovers of the ingot and the remaining material comprising the button and sprue are usually discarded. Apparently, it is more cost effective for laboratories to reuse what is often wasted material to press multiple restorations, thereby reducing the quantity of wasted material. In addition, repressing leftover material will reduce treatment expenses for the patient and preserve environmental resources.

The concern of repressing leftovers of discarded lithium disilicate to fabricate more restorations was raised. AlBakry and Chung evaluated repressing IPS Empress 2 and showed contradictory results [10,11]. Gorman et al. [12] reported no significant difference in biaxial flexural strength and fracture toughness after repressing IPS e.max three times. In contrast, Tang et al. [13] found significant differences in three-point flexural strength, fracture toughness, and hardness after a single repressing of IPS e.max. The homogeneity of the material affected the marginal gap and fracture resistance when mixing a new ingot with different concentrations of leftover IPS e.max Press [14]. 

Thus, a definite answer to the question of whether multiple pressing may alter the mechanical properties, esthetics, and microstructure of the repressed material is still lacking, thereby prompting further investigation. In consequence, the aim of this study was to test the flexural strength, microhardness, color stability, and surface topography of lithium disilicate glass ceramics after three consecutive heat-pressed cycles. The null hypothesis is that lithium disilicate (IPS e.max Press) can be reused after repressing without adversely affecting the mechanical and optical properties.

## 2. Materials and Methods

### 2.1. Specimen Preparation

All specimens were fabricated using the lost wax technique. Bar and disk wax patterns (Renfert, Hilzingen, Germany) were heated and applied into silicone molds with two shapes: a bar shape measuring 24.5 mm × 4.5 mm × 2.5 mm, and a disk shape measuring 8.5 mm diameter × 1.5 mm thickness. Then, the specimens were retrieved from the mold and sprue was added, invested, and the wax burned out in a conventional furnace. A lithium disilicate ingot (IPS e.max Press, Ivoclar Vivadent, Schaan, Liechtenstein) shade (A1) low translucency was selected. Then, the ingots were plasticized and with an alumina plunger were first heat-pressed into an investment mold. The process was performed using a press furnace under the following conditions: stand-by temperature 700 °C; press temperature 920 °C; and holding time 25 min (Programat EP3010, Ivoclar Vivadent, Schaan, Liechtenstein). After pressing, the specimens were divested, and the sprue, button portions, and remaining ingot were separated. Then, the specimens were finished with finishing burs (Diapol kit, Palm Coast, FL, USA) and polished with silicon carbide sandpaper up to 2000 grit using a water-cooled polishing machine (PlanarMet 300, Buehler, Lake Bluff, IL, USA). The second and third heat-pressed specimens were fabricated from the leftovers of the previous pressing cycles. A total of 36 bar-shaped and 15 disk-shaped specimens were divided into three groups according to the number of pressing cycles (n = 12 bars and n = 5 disks): P1: first press (control), P2: second press, and P3: third press. The final dimensions of the bar specimens were 24 × 4 × 2 mm, following the guidelines of ISO 6872-2015 used to evaluate flexural strength for ceramic materials. The disk specimens measured 8 mm in diameter and 1 mm in thickness and were used for the evaluation of color stability, microhardness, and surface topography.

### 2.2. Flexural Strength Testing

**Figure 1:**
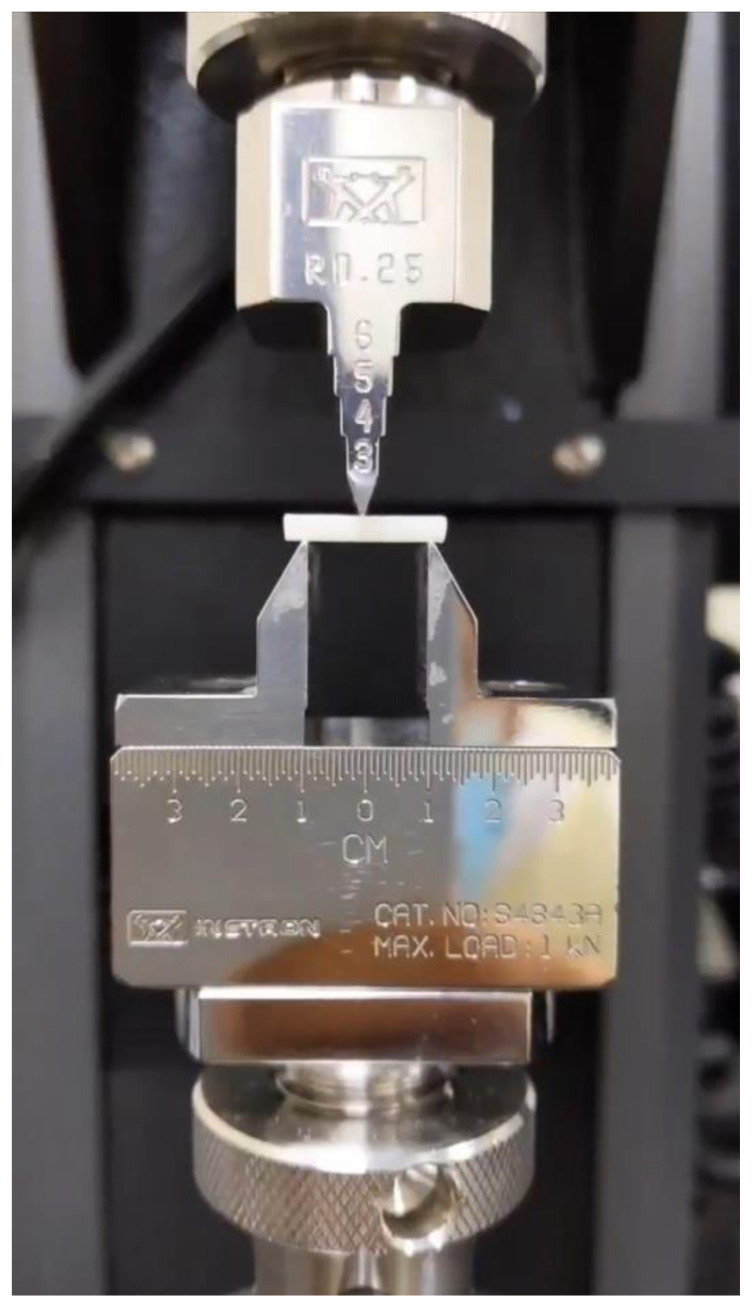
Specimen seated on universal testing machine for testing flexural strength.

Ten bars from each group were tested in a three-point bending assembly attached to a universal testing machine (Multitest 2.5-i, Mecmesin, West Sussex, UK). The specimens were loaded until fracture using a 500 N load cell and crosshead speed 1 mm/min. The flexural strength (FS) was calculated using the formula FS = 3Fl/2bd^2^ following the standard ISO 6872-2015, where F is the fracture load (N), l is the test span (20 mm), b is the bar width (4 mm), and d is the bar thickness (2 mm). The mean for the FS (MPa) of each group was calculated (Figure 1).

### 2.3. Color Stability Evaluation

The color shades of the different press groups were measured using a spectrophotometer (CE7000A, X-rite, Grand Rapids, MI, USA) following the (CIE) L*a*b* system. The machine uses pulsed xenon with a spectral range of 360 nm to 750 nm. This illumination conditions the sample to approximate illuminant D65. Calibration was done with the provided tiles and repeated every session. Each specimen was seated with approximately 10 degrees to face a small aperture (3 × 8 mm) and covered by a black arm to mask all ambient lights. The light source was then pulsed three times toward the sample and the average color measurements were recorded. The L* parameter corresponds to the degree of lightness and darkness, whereas the a* and b* coordinates correspond to red–green and yellow–blue, respectively.

After recording the color coordinates of all groups, the data for each group were compared to those of the other two groups, resulting in three combination pairs (P1–P2, P1–P3, and P2–P3) for each coordinate (L*, a*, and b*). Additionally, the overall color change ∆E_1–2_ (P1 vs. P2) and ∆E_1–3_ (P1 vs. P3) were calculated to evaluate the overall color change from the baseline to the second and third pressing cycles, respectively. ∆E was calculated using the following formula:

$$∆E=\sqrt{\Delta L^{2}+\Delta a^{2\;}+\Delta b^{2}}$$

where ∆L, ∆a, and ∆b are the differences between baseline data P1 and the respective values after repressing with each group P2 and P3.

### 2.4. Vickers Hardness Test

The specimens used for color stability measurement were then reused for the Vickers hardness test. The specimens were stabilized flat on the platform of the hardness tester (Wilson VH1150, Buehler, Chicago, IL, USA) and five measurements were performed for each specimen under a 10 N load for an indentation duration of 10 s. The mean HV for each group was calculated using the following formula: HV = 1.8544 P/d^2^, where P is the applied load (kg) and d is the mean of indentations (mm). 

### 2.5. Scanning Electron Microscopy (SEM)

Two specimens from each group were randomly selected from each group for SEM evaluation. They were etched with 9.6% hydrofluoric acid for 6 minutes, rinsed and dried, and then gold sputtered for 120 seconds (SC7260, Quorum, Laughton, East Sussex, UK). The specimens were scanned under SEM (Aura 100, Seron Technologies, Gyeonggi-do, Korea) and evaluated under 3000× and 5000× magnification to evaluate the microstructure and dimensions of lithium disilicate crystals. 

### 2.6. Statistical Analysis

A one-way ANOVA test was used to evaluate the effect of the number of pressing cycles on the flexural strength and hardness using Statistical Package for the Social Sciences software (SPSS version 20, Chicago, IL, USA). An independent specimens *t*-test was used to evaluate the effect of pressing cycles on the color change of different coordinates and ∆E. All tests were conducted at a significance level of *p* = 0.05.

## 3. Results

### 3.1. Flexural Strength and Vickers Hardness

The data from the flexural strength and Vickers hardness tests are presented in Table 1. The mean (SD) flexural strength values are 269.85 ± 30.48 MPa, 266.66 ± 27.12 MPa, and 253.16 ± 22.18 MPa for groups P1, P2, and P3, respectively. The one-way ANOVA test showed no significant difference in flexural strength between all groups (*p* = 0.283). The mean Vickers hardness values of the groups are P1 = 570.07 ± 20.63 HV, P2 = 563.32 ± 19.08 HV, and P3 = 554.97 ± 15.74 HV. The one-way ANOVA test showed no statistical difference between all groups (*p* = 0.220).

### 3.2. Color Stability (∆E)

Table 2 presents the mean and SD of L*, a*, and b* coordinates of all groups. Overall, repressing led to a reduction of L* and b* values and an increase of a* values, indicating a shift toward darker, bluish, and reddish samples. Table 3 shows the results of the independent specimens t-test of the effect of repressing on color coordinates. 

Significant color changes were reported in all color coordinates between the pairs P1–P2 and P1–P3, whereas no changes were recorded in any of the color coordinates for P2–P3 (*p* < 0.05). The mean color change (∆E) values after the second press (P1 vs. P2) and the third press (P1 vs. P3) were ∆E_1–2_ = 2.04 ± 1.27 and ∆E_1–3_ = 2.14 ± 0.77, respectively. There was no significant difference between ∆E_1–2_ and ∆E_1–3_ (*p* = 0.887). Table 4 represents the overall color change (∆E) from the baseline.

### 3.3. Scanning Electron Microscopy (SEM)

**Figure 2:**
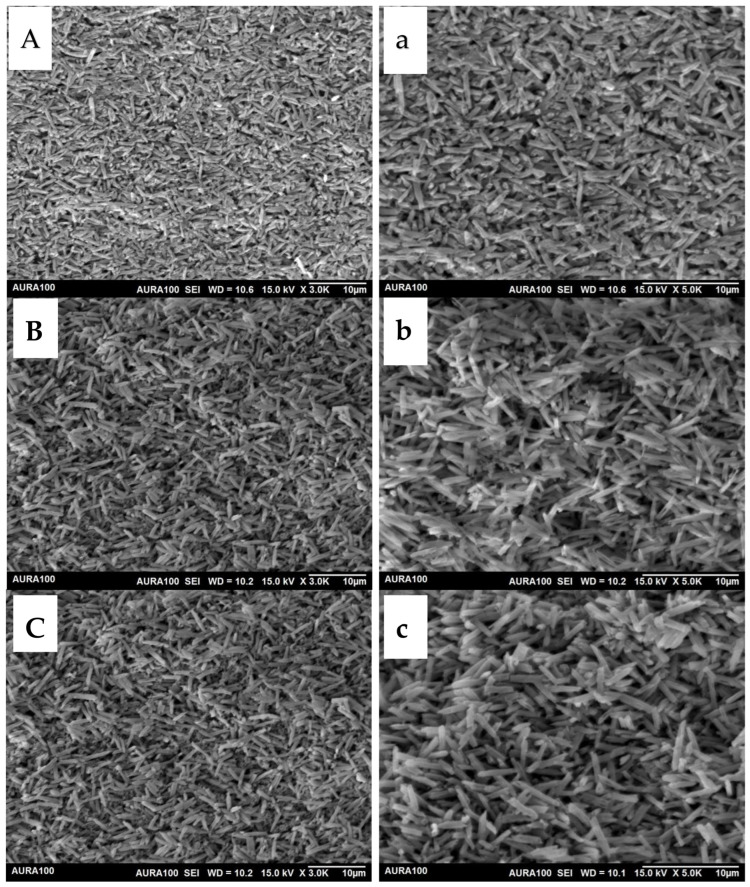
Scanning electron microscope images of each group, under 3000× (left column) and 5000× (right column) magnifications. The first press group (**A**,**a**) show closely packed and interlocked lithium disilicate crystals. The second (**B**,**b**) and third press groups (**C**,**c**) show larger and less densely packed lithium disilicate crystals.

Under 3000× magnification, the SEM image of the first press showed needle-like interlocked lithium disilicate crystals evenly distributed within the glassy matrix (Figure 2A). The lithium disilicate crystals appear densely packed with no specific orientation. After the second and third pressing cycles (Figure 2B,C), the same overall microstructure was seen except that the crystals appeared larger and less packed. Under 5000× magnification, it was possible to quantify the dimensions of the crystals. After the first press, the crystals averaged about 0.3–0.5 µm in diameter (Figure 2a) and 1–3 µm in length compared to 0.4–0.8 µm in diameter and 2–6 µm in length for the second and third press, respectively, resulting in a more blunt-end appearance with a higher aspect ratio (Figure 2b,c). 

## 4. Discussion

This study investigated the effect of the multiple repressing of IPS e.max Press on the flexural strength, microhardness, color stability, and microstructure of the material. After repressing the same material twice, the morphological microstructure showed larger and less densely packed lithium disilicate crystals. However, the flexural strength and microhardness were not adversely affected. Additionally, the overall color parameter ∆E did not show any perceptible change. Thereby, the null hypothesis that lithium disilicate (IPS e.max Press) can be repressed without negatively affecting mechanical and optical properties was accepted. 

In the present study, the mean value of flexural strength for the first press, 269 ± 30.48 MPa, was consistent with previous studies that reported mean values ranging from 239 MPa to 281 MPa [10,12,15,16,17]. However, it was below the values of flexural strength claimed by the supplying manufacturer and reported by others (300–400 MPa) [4,13,18,19].

Our results showed that for up to two repressing cycles, the values of flexural strength did not change significantly. In agreement with these results, Albakry et al. reported a non-significant reduction of flexural strength from P1 (340 MPa) to P2 (325 MPa) [4]. Similarly, Gorman et al. reported a slight elevation followed by reduction of strength in P1 (243 MPa), P2 (252 MPa), P3 (225 MPa), and P3 to P4 (214.8) [12]; however, they were insignificant. Interestingly, Chung et al. reported a significant elevation in strength from P1 (281 MPa) to P2 (365 MPa) after repressing [10]. On the contrary, Tang et al. [13] reported a significant reduction of strength from P1 (354 MPa) to P2 (247 MPa) after repressing. The use of different testing methodology, including using biaxial flexural strength tests [18] and annealing, could lead to the higher flexural strength reported in these studies in comparison to the present study [20].

After repeated pressing, the crystals are subjected to redistribution and growth under the effect of high temperature and pressure [21]. In the current study, the SEM images of the first press showed densely packed lithium disilicate crystals along the glassy matrix. Further pressing revealed less densely packed lithium disilicate crystals with obvious enlargement (Figure 2b,c). The small spaces observed in the SEM images of the repressed materials could be due to precipitates of the lithium orthophosphate phase [22]. They are small crystals <0.3 µm in diameter present in the glassy matrix and have a higher etching rate than the lithium disilicate phase. The crystal enlargement of repressed lithium disilicate is a consistent finding among all SEM observations of repressed lithium disilicate ceramic studies [4,12,13]. Gorman et al. [12] reported an average grain width and length of 0.69 μm and 4.19 μm, respectively, after repeated pressing of IPS e.max Press with a linear grain growth with the increasing number of pressing cycles [23]. Albakry et al. [4] reported grain elongation from 3–5 µm after the first press to approximately 7.5–8.5 µm after repressing Empress 2 lithium disilicate glass ceramic [23]. Tang et al. [13] reported the observation of wider, longer rods, with the disappearance of the sharp ends of the needle-like crystals. The enlargement of the crystals was attributed to the Ostwald ripening phenomenon, which involves the dissolution of the smaller particles and deposition of larger ones, thereby minimizing the surface-to-area ratio [4,24]. 

The present study also evaluated the microhardness values of the repressed lithium disilicate glass ceramics. The initial microhardness values of the first press (570.07 ± 20.63) was in the range reported by the manufacturer of 550 HV and the values reported by Gorman et al. of 548 ± 28.68 HV [12,25]. In the current study, repeated pressing led to a slight decline in the microhardness values in P2 (563.32 ± 19.08) and P3 (554.97 ± 15.74), with no statistically significant difference. The results of the current study agree with the results of Gorman et al., who reported microhardness values of P2 (559 ± 8.68) and P3 (526 ± 49.13) [12].

The firing temperature and number of cycles are influencing factors for the color stability, which is crucial for the success of esthetic restoration [26]. The results showed a significant change in the values of all color coordinates (a*, b*, and L*) between the baseline and the repressed materials. However, no difference was observed between the repressed materials for the second or third time. The fact that groups P2 and P3 had similar color parameters may confirm the relationship between the color and the microstructure of lithium disilicate crystals, where larger crystals were obvious in those groups. Despite the significant difference of the values of the color coordinates, ∆E values of ∆E_1–2_ = 2.04 and ∆E_1–3_ = 2.14 after the second and third pressing cycles, respectively, indicate the acceptable color stability of the repressed materials. In the dental literature, it is widely acknowledged that ΔE values below 2.7–3.3 are clinically acceptable [27,28,29]. The ΔE values of the current study were lower than those reported by ElNaggar et al. [30], who reported a ΔE value of 3.78.

After repressing, topography analysis showed obvious larger and less densely packed crystals of lithium disilicate. This surface change might influence the bond strength of resin cement to the restoration. Thus, one of the limitations of this study was the evaluation of the bond strength of resin cement to the repressed samples. Another limitation is that the influence of multiple repressing on the translucency of the restoration was not investigated.

## 5. Conclusions

According to the findings of this in vitro study, the following conclusions can be drawn:

1. There was no significant difference in the mechanical properties (flexural strength and Vickers microhardness) between the first pressed lithium disilicate group (IPS e.max Press) and the repressed groups.
2. Although the repressed groups tended to be darker and more bluish, the ΔE values after two repeated pressing cycles were within the clinically acceptable range.
3. The SEM images showed that the first press group presented packed interlocked crystals of lithium disilicate, while the repressed groups showed obvious larger and less closely packed crystals.
4. These results may support the reuse of pressed lithium disilicate for economical purposes, but further clinical evaluation should be conducted to confirm these findings.

## Figures and Tables

**Table 1:** Flexural strength and Vickers hardness mean values (±SD).

	1st Press	2nd Press	3rd Press	*p*-Value
Flexural Strength (MPa)	269.85 ± 30.48	266.66 ± 27.12	253.16 ± 22.18	0.283
Vickers Hardness (HV)	570.07 ± 20.63	563.32 ± 19.08	554.97 ± 15.74	0.220

**Table 2:** Color coordinates of baseline (first press) and repressed groups.

Coordinate	1st Press	2nd Press	3rd Press
L	66.10 ± 0.50 *	63.96 ± 0.77	64.0 ± 0.42
a	−1.72 ± 0.05 *	−1.58 ± 0.11	−1.58 ± 0.06
b	2.50 ± 0.22 *	1.83 ± 0.25	2.16 ± 0.16

* Statistically Significant at *p <* 0.05.

**Table 3:** Two-sample *t*-test of color coordinate pairs in relation to pressing times.

Pair	*t*-Value	Degree of Freedom	T Critical Value	*p*-Value
L1–L2	5.24	7	2.37	0.001
L1–L3	7.19	8	2.30	<0.001
L2–L3	−0.11	6	2.45	0.907
a1–a2	−2.52	6	2.45	0.048
a1–a3	−3.75	8	2.30	0.006
a2–a3	0.00	6	2.45	1.000
b1–b2	4.58	8	2.30	0.002
b1–b2	3.23	7	2.37	0.014
b2–b3	−2.17	7	2.37	0.06

**Table 4:** Color change after second press (P1–P2) and third press (P2–P3) cycles.

	1st Press–2nd Press	1st Press–3rd Press	*t*-Value	Degree of Freedom	*p*-Value
∆E	2.04 ± 1.27	2.14 ± 0.77	−0.15	8	0.887
